# Metabolomics of Breast Cancer Using High-Resolution Magic Angle Spinning Magnetic Resonance Spectroscopy: Correlations with 18F-FDG Positron Emission Tomography-Computed Tomography, Dynamic Contrast-Enhanced and Diffusion-Weighted Imaging MRI

**DOI:** 10.1371/journal.pone.0159949

**Published:** 2016-07-26

**Authors:** Haesung Yoon, Dahye Yoon, Mijin Yun, Ji Soo Choi, Vivian Youngjean Park, Eun-Kyung Kim, Joon Jeong, Ja Seung Koo, Jung Hyun Yoon, Hee Jung Moon, Suhkmann Kim, Min Jung Kim

**Affiliations:** 1 Department of Radiology, Research Institute of Radiological Science, Severance Hospital, Yonsei University College of Medicine, Seoul, Republic of Korea; 2 Department of Chemistry and Chemistry Institute for Functional Materials, Pusan National University, Busan, Republic of Korea; 3 Department of Nuclear Medicine, Severance Hospital, Yonsei University College of Medicine, Seoul, Republic of Korea; 4 Department of Radiology, Samsung Medical Center, Sungkyunkwan University School of Medicine, Seoul, Republic of Korea; 5 Department of Surgery, Gangnam Severance Hospital, Yonsei University College of Medicine, Seoul, Republic of Korea; 6 Department of Pathology, Severance Hospital, Yonsei University College of Medicine, Seoul, Republic of Korea; Fondazione IRCCS Istituto Nazionale dei Tumori, ITALY

## Abstract

**Purpose:**

Our goal in this study was to find correlations between breast cancer metabolites and conventional quantitative imaging parameters using high-resolution magic angle spinning (HR-MAS) magnetic resonance spectroscopy (MRS) and to find breast cancer subgroups that show high correlations between metabolites and imaging parameters.

**Materials and methods:**

Between August 2010 and December 2013, we included 53 female patients (mean age 49.6 years; age range 32–75 years) with a total of 53 breast lesions assessed by the Breast Imaging Reporting and Data System. They were enrolled under the following criteria: breast lesions larger than 1 cm in diameter which 1) were suspicious for malignancy on mammography or ultrasound (US), 2) were pathologically confirmed to be breast cancer with US-guided core-needle biopsy (CNB) 3) underwent 3 Tesla MRI with dynamic contrast-enhanced (DCE) and diffusion-weighted imaging (DWI) and positron emission tomography-computed tomography (PET-CT), and 4) had an attainable immunohistochemistry profile from CNB. We acquired spectral data by HR-MAS MRS with CNB specimens and expressed the data as relative metabolite concentrations. We compared the metabolites with the signal enhancement ratio (SER), maximum standardized FDG uptake value (SUV max), apparent diffusion coefficient (ADC), and histopathologic prognostic factors for correlation. We calculated Spearman correlations and performed a partial least squares-discriminant analysis (PLS-DA) to further classify patient groups into subgroups to find correlation differences between HR-MAS spectroscopic values and conventional imaging parameters.

**Results:**

In a multivariate analysis, the PLS-DA models built with HR-MAS MRS metabolic profiles showed visible discrimination between high and low SER, SUV, and ADC. In luminal subtype breast cancer, compared to all cases, high SER, ADV, and SUV were more closely clustered by visual assessment. Multiple metabolites were correlated with SER and SUV in all cases. Multiple metabolites showed correlations with SER and SUV in the ER positive, HER2 negative, and Ki-67 negative groups.

**Conclusion:**

High levels of PC, choline, and glycine acquired from HR-MAS MRS using CNB specimens were noted in the high SER group via DCE MRI and the high SUV group via PET-CT, with significant correlations between choline and SER and between PC and SUV. Further studies should investigate whether HR-MAS MRS using CNB specimens can provide similar or more prognostic information than conventional quantitative imaging parameters.

## Introduction

Breast cancer encompasses a heterogeneous group of diseases with various histological differentiations, clinical courses, and responses to treatment. Along with early detection, identifying reliable markers to improve diagnostic accuracy and prognosis is important in the treatment of breast cancer. In addition to traditional parameters such as tumor size, tumor grade, and lymph node status, several molecular markers are now used to classify breast cancers into subgroups and to predict clinical outcomes [1, 2]. The most frequently used molecular markers are based on immunohistochemical (IHC) profile expression, such as expression of the estrogen receptor (ER), progesterone receptor (PR), human epidermal growth factor receptor 2 (HER2), and Ki-67 [3]. High-resolution magic angle spinning (HR-MAS) magnetic resonance spectroscopy (MRS) has been recently suggested as a promising tool in the diagnosis and characterization of breast cancer [4–8]. The technique can be used to measure multiple cellular metabolites simultaneously and to provide a vast amount of information on biochemical composition by analyzing tissue samples. Recent studies using HR-MAS MRS have found different concentrations of choline-containing compounds in breast cancer tissue and these different distributions have been correlated with clinicopathological parameters that predict tumor aggressiveness [7–9]. A recent study suggested that HR-MAS MRS using core-needle biopsy (CNB) specimens could predict tumor aggressiveness prior to surgery because several molecular markers significantly correlated with histologic prognostic factors [4].

Morphological and functional parameters that are influenced by tumor biology are used to study and compare imaging techniques. Dynamic contrast-enhanced (DCE) MRI is a well-established technique for monitoring contrast enhancement kinetics that reveal the characteristics of tumor microvasculature. For example, early enhancement and the washout kinetic curve have been correlated with high histologic grades or ER negativity [10, 11]. Diffusion-weighted imaging (DWI) represents the biological character of the tumor and apparent diffusion coefficient (ADC) values are used to obtain information on tissue cellularity. A previous study reported that a lower ADC value was related to the positive expression of ER and negative expression of HER2 [12]. A lower ADC was also associated with the positive expression of ER, and PR, increased Ki-67, and increased microvascular density in breast cancer [13]. 18F-fluorodeoxygluxose (FDG) positron emission tomography-computed tomography (PET-CT) reflects glucose metabolism and uses a standardized FDG uptake value (SUV) for tumor characterization. SUV correlates with histological grade and expression of ER and PR [14]. Koo et al. reported higher SUV values in triple negative and HER2 positive cancers than in the luminal A subtype of breast cancer [15]. Because quantitative parameters in conventional imaging modalities relate to prognostic factors in breast cancer, it is possible to predict prognosis by establishing correlations between quantitative imaging parameters and breast cancer metabolites. There have been some attempts to do this with in vivo MRS studies. Total choline levels using in vivo MRS were well correlated with SUV and prognostic parameters such as nuclear grade, ER and triple negative status [16], and pharmacokinetic parameters that represent washout in DCE MRI [17, 18]. To our knowledge, no previous studies have compared HR-MAS MRS with other breast imaging modalities and clinicopathological prognostic factors to demonstrate surrogate biomarkers in breast cancer.

The purpose of our study was to investigate the relationship between breast cancer metabolites and conventional quantitative imaging parameters using HR-MAS MRS and to find breast cancer subgroups that have high correlations between metabolites and imaging parameters.

## Materials and Methods

### Patients

This study was approved by the institutional review board of Yonsei University College of Medicine, and we obtained written informed consent from each patient prior to study commencement.

Between August 2010 and December 2013, we included 53 female patients (mean age 49.6 years; age range 32–75 years) with a total of 53 breast lesions assessed by the Breast Imaging Reporting and Data System. The patients were enrolled under the following criteria: breast lesions larger than 1 cm in diameter that 1) were suspicious for malignancy on mammography or ultrasound (US), 2) were pathologically confirmed to be breast cancer with US-guided CNB, 3) underwent 3 Tesla MRI with DCE and DWI and PET-CT, and 4) had an attainable IHC profile from CNB.

### US-guided Core-needle Biopsy and Sample Preparation

US-guided CNBs were performed with a 14-gauge dual action semiautomatic core biopsy needle (Stericut with coaxial guide; TSK Laboratory, Tochigi, Japan) by one of four radiologists (with 6–13 years of experience). The mean number of tissue samples obtained by US-guided CNB for each lesion was six (range 5–8). All samples except for one core sample of each lesion were used for pathologic diagnosis and IHC analysis. For HR-MAS MRS, one core tissue sample was put in a cryogenic vial and immersed in liquid nitrogen immediately after biopsy. Samples were stored at –70°C for one to five months prior to HR-MAS MRS.

### Histopathologic Analysis

All 53 lesions were pathologically diagnosed as malignant by CNB prior to treatment. Information about pathologic variables, including histologic grade and ER, PR, HER2, and Ki-67 status, was obtained with the CNB specimen. Fifty patients underwent surgery, and we compared their final histopathologic results. All tissues were fixed in 10% buffered formalin and embedded in paraffin. Each section was stained with hematoxylin-eosin (H&E) for microscopic examination by experienced pathologists. The histologic grade of each tumor was determined using the modified Bloom-Richardson classification [19]. Another section was stained immunohistochemically for ER, PR, and HER-2/neu using commercially available antibodies for ER (Thermo Scientific, Fremont, CA, USA), PR (Dako, Glostrup, Denmark), c-erbB-2 (Dako), p53 (Dako), and Ki-67 (Novocastra, Newcastle, UK). We defined ER and PR positivity as the presence of 10% or more positively stained nuclei in 10 high-power fields. The intensity of HER-2 staining was semi-quantitatively scored as 0, 1+, 2+, or 3+. We considered tumors scored as 3+ to be HER2 positive cases and tumors scored from 0 to 1+ to be negative cases. Borderline cases (2+) required further investigation using fluorescence in situ hybridization to assess gene amplification. We scored the IHC staining of Ki-67 by counting the number of cells with positively stained nuclei and expressed results as a percentage of the total tumor cells. The cut-point value for positive Ki-67 was 14% [20].

### MRI Technique

MR examinations were performed using two 3-T MR scanners (TrioTim; Siemens, Erlangen, Germany [MR system 1] / Discovery MR750; GE Medical Systems, Milwaukee, WI, USA [MR system 2]) with 18 and 35 patients, respectively, for each scanner. Imaging was performed with a dedicated phased array breast coil with the patient in the prone position. After obtaining 3-plane localizer images, we obtained axial T2-weighted turbo or fast spin-echo images (TR/TE 4360/82; matrix 512 × 512 pixels, field of view 340 × 340 mm; section thickness 3 mm for MR system 1 and TR/TE 4187/102; matrix 416 × 256 pixels, field of view 320 × 320 mm; section thickness 3 mm for MR system 2) and axial T2 STIR images (TR/TE 4500/76; TI 220 ms for MR system 1 and TR/TE 5000/70; TI 200 ms for MR system 2). Diffusion-weighted images with a 2D spin-echo echo-planar imaging sequence (TR/TE 9100/80 for MR system 1 and TR/TE 6000/68; TI 250ms for MR system 2) were obtained thereafter. We applied diffusion-weighted gradients in three orthogonal directions and used two b-values: 0,850 (n = 2) and 0,600 (n = 67).

After DWI MRI, we performed T1-weighted DCE MRI, including one pre-contrast acquisition and six post-contrast bilateral axial acquisitions (TR/TE 280/2.6; matrix 512 × 343 pixels, field of view 340 × 340 mm; section thickness 3mm, no intersection gap for MR system 1 and VIBRANT-Flex Dynamic imaging (TR/TE 4100/1700); matrix 280 × 512 pixels; flip angle 12°; field of view 320 × 320 mm; section thickness 3mm, no intersection gap for MR system 2). A gadolinium-based contrast agent (Dotarem; Guerbet, Paris, France/ Magnevist; Berlex Laboratories, Wayne, NJ, USA/Gadovist; Bayer Schering Pharma, Berlin, Germany) was injected into an antecubital vein at a dose of 0.2 cc per kilogram of body weight at a rate of 2 mL/sec using an automated injector and followed by a 20-mL saline flush. Post-contrast images were obtained 20 seconds after the start of contrast material injection for MR system 1 or immediately after contrast material injection with no time delay for MR system 2. The acquisition time for each post-contrast series was 60 seconds and 75 seconds for MR systems 1 and 2, respectively. Thus, six post-contrast images were obtained at approximately 90, 150, 210, 270, 330, 390 seconds for MR system 1 and at 90, 165, 240, 315, 390, 465 seconds for MR system 2 after the start of contrast material administration. Temporal sampling of the center of the k-space for the post-contrast series occurred at approximately 45, 105, 165, 225, 285, 345 seconds for MR system 1 and at 45, 120, 195, 270, 345, 420 seconds for MR system 2. For all dynamic studies, we generated subtraction images. Menstrual cycles were not considered when scheduling MR imaging examinations.

### PET-CT Imaging Technique

PET was performed using a PET/CT system (Discovery STE; GE Medical Systems, Milwaukee, WI, USA). All patients fasted for at least 5 hours before PET imaging. Image acquisition started 1 hour after intravenous administration of FDG (3.7 MBq/kg body weight). CT scans from the brain to the pelvis were performed immediately before the PET scans using a multi-detector spiral CT scanner (3.75 mm slice thickness, pitch of 1.75, 120 keV, and 30–200 mA depending on the patient’s total body mass). We performed whole-body PET scans, covering an area identical to that covered by the CT scan. All PET/CT images were interpreted at the workstation (Advantage Windows workstation; GE Healthcare).

### HR-MAS MRS Experiments

We measured HR-MAS MR spectra of the tissue samples with a nuclear magnetic resonance (NMR) spectrometer (VNMRS 600, Agilent, Walnut Creek, CA, USA) operating at a proton NMR frequency of 600.167 MHz (14.09T). Temperature was set to 19°C. Each experiment took approximately 20 minutes. Frozen samples were cut and weighed on ice in the NMR laboratory, and placed in an HR-MAS nanotube (Agilent). The total volume of the sample cell was 40μl, and an average of 8.4mg core-biopsy samples were placed in the cell with the remaining volume filled with D_2_O containing 2mM of trimethylsilyl propionic acid (TSP). The probe was an inverse-detection type equipped with a single Z-gradient coil.

The CNB tissue samples were analyzed using the Carr-Purcell-Meiboom-Gill pulse sequence to impose a T2 filter. All data were collected at a spinning rate of 2 kHz. The spectral acquisition parameters were as follows: 19.231 K complex data points, 9615.4 Hz sweep width, 2.0 s acquisition time, 1.0 s relaxation delay, 1.5 s saturation time, 256 number of transients, 10 receiver gain, and total acquisition time of 16 min 18 sec. Each free induction decay signal was processed and analyzed using Chenomx NMR Suite 7.1 professional software (Chenomx Inc., Edmonton, Canada). Post-processing consisted of Fourier transformation, phasing, and baseline correction. Data quantification was performed by comparing the integrated TSP signal with the signal of interest in the tumor spectrum and the relative concentrations were recorded.

### Image Review and Quantitative Analysis

All MRI examinations were subsequently processed by CADstream (Confirma, Inc., Kirkland, WA, USA), a commercially available computer-aided detection system. To assess lesion kinetics, we assessed the whole series of DCE MR images. We gave the reviewers information about tumor location for a more consistent quantitative analysis. To analyze the time intensity curve, we manually drew regions of interest (ROIs) around the lesions, avoiding necrotic or cystic components. The kinetic type of lesions were categorized as persistent, plateau, or washout based on images obtained in the delayed enhancement phase [21]. We calculated the signal enhancement ratio (SER) of the tumor using the following equation [22]: SER = (S1-S0)/ (S2-S0) where S0, S1, S2 represent the signal intensity on pre-contrast (before contrast material administration), early post-contrast (90 seconds after), and delayed post-contrast (395–435 seconds after) images, respectively.

The CADstream commercial software (Merge Healthcare, Milwaukee, WI, USA) automatically calculated an ADC map using the signal intensity within the manually drawn ROI. The ROIs were drawn at the most representative slice of the corresponding location and in accordance with the size of the tumor, as reflected in the DCE MRI analysis. Apparent necrotic or cystic components were avoided by referring to the DCE MRI and T2-weighted images. We calculated the ADC value as the mean of the voxels within the ROI of the tumor. The multiple b-value method calculated the ADC using a least-square exponential fitting of all b-value data within the defined range of b-values. All diffusion-weighted images were first divided by the lowest b-value image, and the resultant images were based on relative signal intensities. For each pixel in the image, we performed a linear regression to fit a straight line to the natural logarithm of the relative signal intensities over the respective b-values.

Two radiologists reviewed and interpreted all the PET-CT and MRI images in consensus. Lesions were analyzed semi-quantitatively using the SUV max, defined as the maximum tissue concentration of FDG (kBq/ml) in the structure delineated by the ROI divided by the activity injected per gram body weight (kBq/g). The maximum SUV for each visible breast cancer was automatically recorded by drawing a circular 3-D ROI over the largest area with abnormal FDG uptake in the breast cancer lesion to cover the entire tumor volume. The SUV max of the liver was measured as reference for comparison.

### Data and Statistical Analysis

We collected patients’ clinicopathological data by reviewing medical records. Clinicopathological variables included the pathologic type of each tumor, lymph node metastasis at the time of diagnosis, status of ER, PR, HER2, and Ki-67 expression, and triple negativity (Table 1). Tumor size was defined as the size reported on the final pathologic results (n = 27), except for cases in which the patient received neoadjuvant chemotherapy before surgery (n = 23) or did not undergo surgery (n = 3). In those patients, tumor size was defined as the size measured with MRI.

**Table 1:** Clinicopathological data of the 53 patients with 53 malignant breast lesions in this study.

Clinicopathological variables	Patients (%)
Histologic grade	
Low (Grade 1–2)	29 (54.7)
High (Grade 3)	17 (32.1)
N/A	7 (13.2)
Tumor size	
≤2 cm	25 (47.2)
>2 cm	28 (52.8)
ER status	
Negative	17 (32.1)
Positive	36 (67.9)
PR status	
Negative	37 (69.8)
Positive	16 (30.2)
HER2 status	
Negative	41 (77.4)
Positive	12 (22.6)
Ki-67 status	
Low (<14%)	22 (41.5)
High (≥14%)	29 (54.7)
N/A	2 (3.8)
Triple status	
Negative	12 (22.6)
Positive	1 (1.8)

N/A: not available.

Spectral data acquired by HR-MAS MRS were expressed as relative metabolite concentrations. These metabolites were compared with SER, SUV max, ADC, and histopathologic prognostic factors for correlation. We performed all analyses using IBM SPSS Statistics 21.0 (IBM SPSS Statistics for Windows, Version 21.0; Armonk, NY, USA). An arbitrary decision was made to use median values to divide high and low groups for each imaging parameter category. The Mann-Whitney U test was performed to compare metabolic data between these groups. We used the Spearman correlation coefficient to show relationships between variables. P-values less than 0.05 were considered significant.

For multivariate analysis of spectral data, we used Matlab (MathWorks, Natick, MA, USA), SIMCA-P 11.0 (Umetrics, Sweden), and Excel (Microsoft, Seattle, WA, USA). We performed partial least squares-discriminant analysis (PLS-DA) to further classify patient groups into subgroups to find correlation differences between HR-MAS spectroscopic values and conventional imaging parameters. We performed additional PLS-DA in luminal subtype breast cancer because multiple breast cancer metabolites showed correlations with SER and SUV in the ER positive, HER2 negative, and Ki67 negative groups. We built class discrimination models until the cross-validated predictability value did not significantly increase to prevent over-fitting of the statistical model. The statistical model was validated by prediction of unknown samples using a leave-one-out analysis [23, 24]. We used an a priori cut-off value of 0.5 to evaluate the prediction results [25]. Signals contributing to group discrimination were identified by an S-plot, and the corresponding HR-MAS MR spectral data were identified using Chenomx (Spectral database; Edmonton, Alberta, Canada) software and a database built in-house.

## Results

The mean tumor size of the 53 breast cancers was 25.8 mm (range, 10–80 mm). The pathological types of the tumors were invasive ductal carcinoma (n = 46), invasive lobular carcinoma (n = 4), invasive carcinoma with ductal and mucinous features (n = 1), invasive carcinoma with ductal and lobular features (n = 1), and mucinous carcinoma (n = 1).

**Fig 1:**
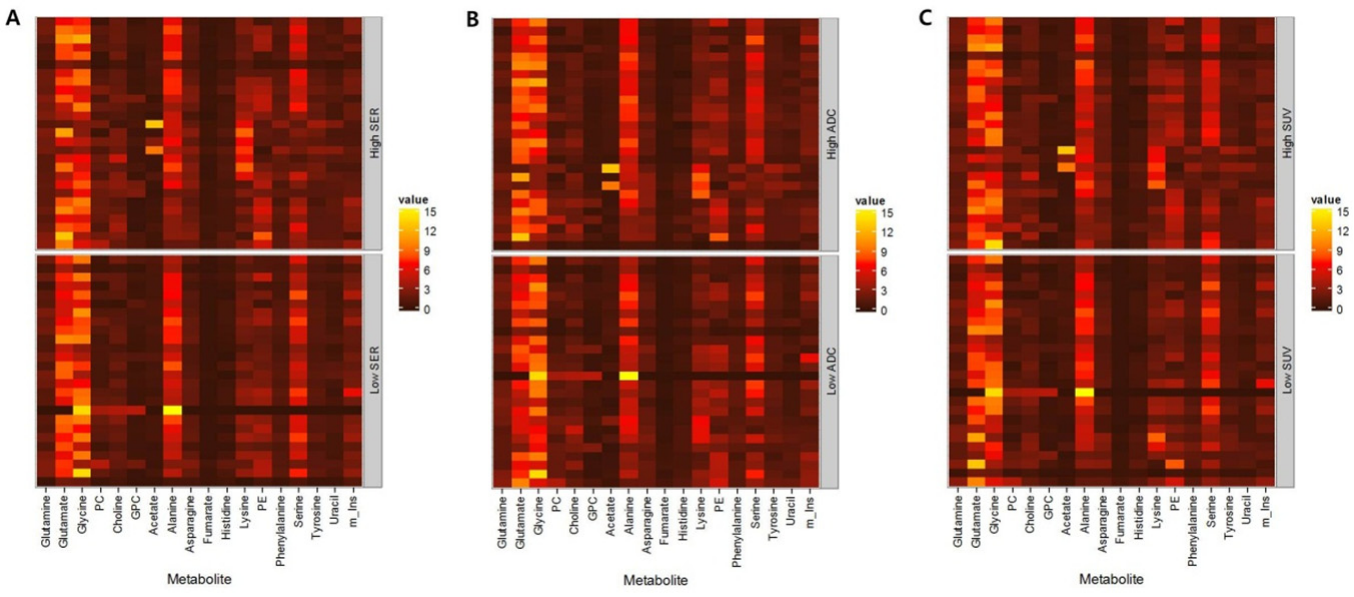
Heat map of HR-MAS MRS metabolites according to the high and low (A) SER, (B) ADC, (C) SUV groups. SER: signal enhancement ratio, SUV: standard uptake value, ADC: apparent diffusion coefficient PC: Phosphocholine, PE: Phosphoethanolamine, GPC: Glycerophosphocholine, m_Ins: myo-Inositol.

The high SER (≥0.96) group showed higher relative metabolite concentrations than the low SER (<0.96) group for asparagine, choline, fumarate, histidine, lysine, phenylalanine, and uracil. The low ADC (<1.38) group showed lower relative metabolite concentrations than the high ADC (≥1.38) group for fumarate and glutamate. The high SUV (≥5.4) group showed higher relative metabolite concentrations than the low SUV (<5.4) group for asparagine, fumarate, PC, PE, and uracil (Fig 1 and S1 Table). SER and SUV showed moderate correlation (r* = 0.445) with each other (P<0.001, Spearman correlation).

**Fig 2:**
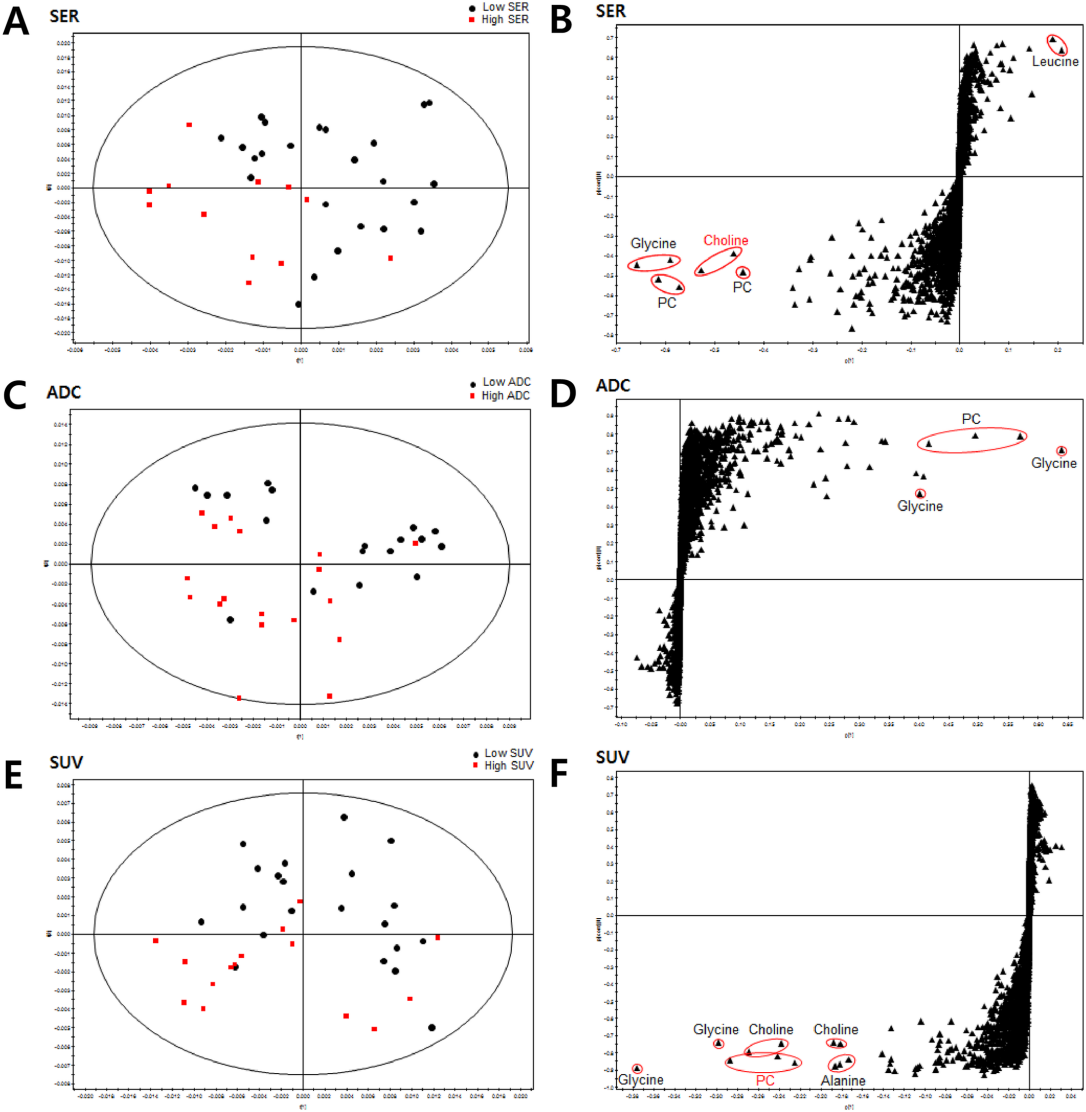
Partial Least Squares-Discriminant Analysis (PLS-DA) score plot and loadings S plot for (A,B) SER, (C,D) ADC, and (E,F) SUV of all cases.

**Fig 3:**
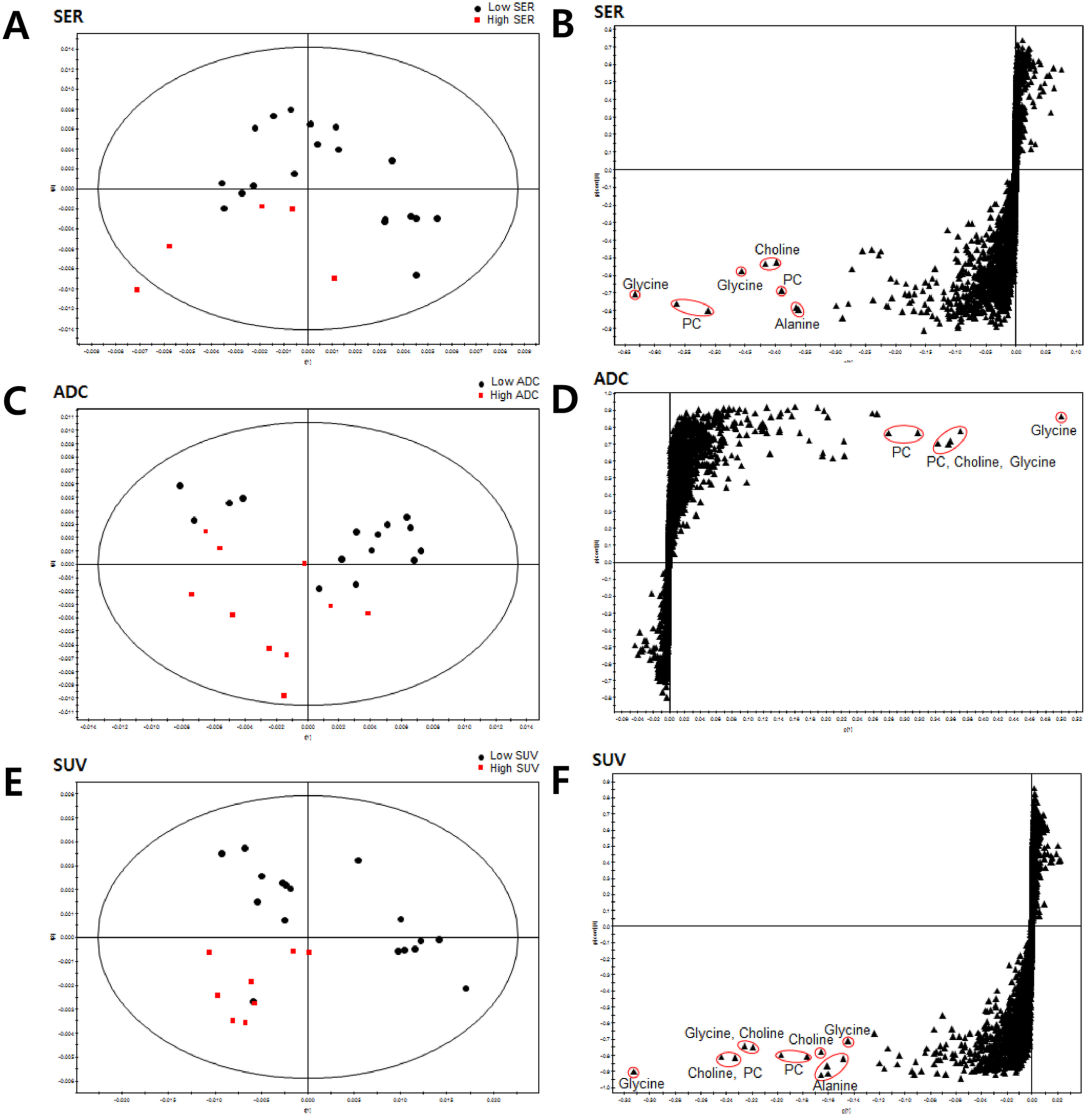
Partial Least Squares-Discriminant Analysis (PLS-DA) score plot and loadings S plot for (A,B) SER, (C,D) ADC, and (E,F) SUV of luminal subtype breast cancer.

We produced PLS-DA separation models with the HR-MAS MR spectral data using the quantitative parameters (SER, ADC, SUV) from conventional imaging. PLS-DA score plots showed visible discrimination by status of high and low SER, SUV, and ADC with some samples crossing over the reference line (Fig 2). In luminal subtype breast cancer, compared to all cases, high SER, ADC, and SUV seemed to be more closely clustered by visual assessment (Fig 3). However, the overall diagnostic performance was not improved except for ADC (Table 2). Corresponding PLS-DA loadings S-plots showed extreme metabolites that contributed to the prediction of high and low groups for SER, ADC, and SUV. Higher levels of PC, choline, and glycine were noted in the high SER group, and a higher level of leucine was found in the low SER group. Higher levels of glycine and PC were noted in the low ADC group. Higher levels of PC, choline, and glycine were noted in the high SUV group. Our PLS-DA prediction model exhibited high diagnostic performance in predicting high and low groups of conventional quantitative imaging parameters (Table 2).

**Table 2:** Diagnostic performance of PLS-DA models in predicting high and low groups of conventional quantitative parameters (SER, ADC, SUV).

	TP	TP+FN	TN	TN+FP	Sensitivity	Specificity	Accuracy
All cases							
SER	10	12	15	24	0.833	0.625	0.694
ADC	12	18	11	18	0.667	0.611	0.639
SUV	11	15	11	21	0.733	0.524	0.611
Luminal subtype							
SER	4	5	12	20	0.800	0.600	0.640
ADC	8	10	11	15	0.800	0.733	0.760
SUV	7	8	8	17	0.875	0.471	0.600

TP: True positive, TN: True negative, FP: False positive, FN: False negative

SER: signal to enhancement ratio, ADC: apparent diffusion coefficient, SUV: standardized uptake value.

As shown in Table 3, we found several metabolite markers via HR-MAS MRS that correlated with SER and SUV with a p value less than 0.05. SER showed positive correlations with asparagine (r* = 0.41), choline (r* = 0.29), fumarate (r* = 0.41), glutamate (r* = 0.30), histidine (r* = 0.44), PE (r* = 0.29), phenylalanine (r* = 0.34), tyrosine (r* = 0.32), uracil (r* = 0.50), and total choline (r* = 0.29). SUV showed positive correlations with asparagine (r* = 0.33), fumarate (r* = 0.41), glutamate (r* = 0.28), lactate (r* = 0.32), PC (r* = 0.30), PE (r* = 0.36), uracil (r* = 0.48), and total choline (r* = 0.28). SUV showed a negative correlation with isoleucine (r* = -0.27).

**Table 3:** Correlation between conventional quantitative parameters (SER, ADC, SUV) and HR-MAS MR spectroscopy values in the 53 breast cancer specimens.

	SER		ADC		SUV	
	r*	p-value	r*	p-value	r*	p-value
Acetate	-0.01	0.963	0.11	0.440	0.02	0.860
Alanine	-0.05	0.738	0.18	0.207	-0.01	0.951
Arginine	0.10	0.462	0.07	0.632	-0.01	0.963
Asparagine	**0.41**	**0.003**	0.13	0.361	**0.33**	**0.016**
Aspartate	0.21	0.133	0.19	0.177	0.17	0.213
Betaine	-0.13	0.364	0.11	0.445	0.13	0.337
Choline	**0.29**	**0.037**	-0.03	0.840	0.17	0.215
Creatine	0.11	0.443	-0.10	0.487	0.15	0.275
Ethanol	0.24	0.083	0.20	0.156	0.05	0.712
Ethanolamine	0.25	0.069	0.18	0.187	0.06	0.654
Fumarate	**0.41**	**0.003**	0.22	0.107	**0.41**	**0.002**
Glucose	0.04	0.766	-0.03	0.830	0.02	0.898
Glutamate	**0.30**	**0.030**	0.26	0.063	**0.28**	**0.042**
Glutamine	0.23	0.090	-0.04	0.750	0.14	0.312
Glycerol	0.17	0.230	-0.02	0.906	0.21	0.136
Glycine	-0.02	0.889	-0.26	0.059	0.07	0.602
Histidine	**0.44**	**0.001**	-0.03	0.844	0.18	0.186
Isoleucine	0.05	0.739	0.03	0.833	**-0.27**	**0.049**
Lactate	0.05	0.731	0.06	0.689	**0.32**	**0.019**
Leucine	0.03	0.833	0.08	0.577	-0.11	0.437
Lysine	0.22	0.119	-0.09	0.519	0.09	0.522
Methionine	0.20	0.149	0.00	0.996	0.00	0.981
PC	0.25	0.075	-0.06	0.667	**0.30**	**0.030**
PE	**0.29**	**0.035**	-0.09	0.526	**0.36**	**0.008**
Phenylalanine	**0.34**	**0.013**	-0.11	0.412	0.05	0.736
Proline	0.14	0.322	-0.09	0.500	0.13	0.345
Serine	-0.07	0.602	-0.09	0.522	-0.09	0.544
Taurine	-0.05	0.699	-0.26	0.055	0.02	0.905
Threonine	0.04	0.778	-0.24	0.086	0.10	0.473
Tyrosine	**0.32**	**0.020**	0.01	0.944	0.08	0.552
Uracil	**0.50**	**0.000**	0.16	0.263	**0.48**	**0.000**
Valine	-0.09	0.515	-0.04	0.754	-0.17	0.229
myo-Inositol	-0.07	0.630	-0.12	0.376	-0.01	0.924
GPC	-0.10	0.459	0.05	0.719	-0.01	0.948
Total choline	**0.29**	**0.035**	-0.075	0.593	**0.28**	**0.039**

r*: Spearman correlation coefficient.

SER: signal enhancement ratio, SUV: standard uptake value, ADC: apparent diffusion coefficient

PC: Phosphocholine, PE: Phosphoethanolamine, GPC: Glycerophosphocholine

Total choline: PC + GPC + choline.

We divided patients into groups by their immunohistochemistry results and compared the SER, ADC, and SUV correlations with metabolites. Multiple metabolites showed correlations with SER and SUV in the ER positive, HER2 negative, and Ki-67 negative groups (S2 Table). The SER of the ER positive group showed positive correlations with asparagine (r* = 0.43), ethanol (r* = 0.34), ethanolamine (r* = 0.40), fumarate (r* = 0.53), glutamine (r* = 0.36), histidine (r* = 0.61), lysine (r* = 0.38), phenylalanine (r* = 0.44), tyrosine (r* = 0.45), and uracil (r* = 0.61). The SUV of the ER positive group showed positive correlations with fumarate (r* = 0.43) and lactate (r* = 0.34). None of the metabolites in the ER negative group showed correlations with SER or SUV. In the HER2 negative group, seven metabolites showed positive correlations with SER, and seven metabolites showed positive correlations with SUV, while one metabolite showed a negative correlation. In the Ki-67 negative group, five metabolites showed positive correlations with SER, while one metabolite showed a negative correlation, and four metabolites showed positive correlations with SUV.

## Discussion

Recently, several studies have focused on HR-MAS MRS to understand breast cancer [4, 7, 8, 26]. HR-MAS MRS can detect more than 30 metabolites in breast cancer tissue [27]. However, these metabolites cannot represent the whole breast tumor. Therefore, it is important to compare HR-MAS MRS results with conventional breast imaging modalities that reflect more complete breast cancer characteristics. Validating the correlations between metabolomics found using HR-MAS MRS and quantitative conventional breast imaging must precede the application of HR-MAS MRS in daily practice for breast cancer diagnosis. In our review of previous literature, we found only a few reports that discussed correlations between different imaging parameters and metabolites in in vivo MRS. The pharmacokinetic parameter k_ep_ in DCE MRI [17, 18] and SUV in PET-CT [16] were correlated with choline levels found with in vivo MRS. Unlike in vivo MRS, ex vivo HR-MAS MRS obtains high-resolution spectra directly from biopsy tissue and allows accurate assessment of correlations between quantitative imaging parameters and metabolites.

We observed higher levels of PC, choline, and glycine in the high SER group in our study. Out of those three metabolites, choline showed a significant correlation with SER (r* = 0.29), which is consistent with previous studies that detect higher total choline concentrations in breast cancer with higher pharmacokinetic parameters (k_ep_) from DCE MRI, suggesting a correlation between choline metabolism and angiogenesis [17, 18]. Total choline compounds were correlated with SER with borderline significance (p = 0.058). Our study results emphasize a more specific relationship between choline and breast cancer angiogenesis compared to previous in vivo MRS studies. Ex vivo HR-MAS MRS can discriminate and quantify PC, GPC, and choline that contribute to the total choline peak in in vivo studies. An elevated level of choline might be associated with increased membrane synthesis due to the ongoing tumor cell replication and angiogenesis required to support tumor growth [17]. Higher contrast washout in DCE MRI correlates with tumor grade [28], histologic grade, and ER negativity [10]. Despite the debate on the relationship between the kinetic parameters of DCE MRI and prognostic factors [29, 30], the SER parameter can sufficiently provide biologic information about angiogenesis [31]. HR-MAS MRS data from biopsied specimens can predict angiogenesis by analyzing the endogenous signaling metabolites that are used to determine tumor prognosis. A previous report also observed a trend toward higher concentrations of choline in poor prognosis breast cancer samples (p = 0.07) using HR-MAS MRS [4]. Therefore, choline could be a promising metabolite in predicting angiogenesis and poor prognosis in breast cancer.

We observed higher levels of PC, choline, and glycine in the high SUV group in our study. PC showed significant correlation with SUV (r* = 0.30). In a previous in vivo MRS study, Tozaki et al. showed a correlation between SUV and total choline levels, a summation of PC, GPC, and free choline [16]. PC, a precursor of cell membrane synthesis, composes a large part of the total choline compound [8]. Therefore, our study correlates well with the previous in vivo study by emphasizing cell-associated alterations in choline metabolism in breast cancer. PC increases during malignant transformation through increased phosphorylation by choline kinase [32, 33]. Also, in a previous ex vivo MRS study, cancer samples that were strongly positive for Ki-67 showed higher concentrations of total choline compound and PC [4]. High SUV levels have been related to poor prognostic markers, showing high relapse and mortality [34, 35]. Therefore, high PC levels might indicate rapid cell turnover in breast cancer and show good correlation with high SUV levels. The SUV max showed positive correlation with lactate (r* = 0.32) and no significant correlations with glucose or alanine. It was previously reported that some breast cancer cell lines showed different rates of lactate uptake and different generation rates of its catabolites (glutamate and alanine) [36]. Another study found that conversion from glucose to lactate and alanine occurred faster in the luminal-like model compared with the basal-like model of cancer despite the fast growth rate of the basal-like model [37]. Lactate showed significant positive correlation with the SUV max in the ER positive, PR positive, HER2 negative and Ki 67 negative groups in our study. This stays in accordance with previous reports that suggest that the tumor growth rate is not necessarily a determinant of glycolytic activity [37]. On the other hand, Cao et al. reported increased lactate levels in the ER negative and PR negative groups [26]. At this time, not many studies regarding lactate levels with molecular types of breast cancers have been published and conclusions based on these studies are still controversial. It has been reported that in vivo lactate MRS imaging has a greater dynamic range than 18F-FDG PET and may be more sensitive in evaluating the aggressive potential of primary breast tumors [38]. Future studies that include larger numbers of patients with each molecular type of breast cancer may demonstrate the correlation between glycolytic activity and tumor aggressiveness. Isoleucine showed negative correlation with SUV in our study. This could not be clearly explained based on previous studies [39]. However, there is a high possibility that this may be due to some complex regulated pathways related to breast cancer. Previous report has suggested that isoleucine degradation was differentially regulated in breast cancer metabolic pathway. Nevertheless, isoleucine degradation could not be associated with oncogenesis and may need further evaluation [39]

DWI is useful in differentiating malignant lesions from benign lesions and provides information about tumor biology and microstructural features. ADC is strongly affected by the architecture of tumors, such as cellular density and stromal features [40]. In our study, we found no significant correlation between metabolites and ADC values, possibly because breast cancer metabolites represent function and ADC values represent structural information. Glycine and PC were suggested as extreme metabolites related to the low ADC group. In many previous studies, a low ADC value has been considered as a promising prognostic factor that identifies highly aggressive breast cancer [41–43]. Although we found no intracellular correlations between ADC and metabolites in this study, we did note high levels of glycine and PC in the low ADC group, which could contribute to poor prognosis.

We observed higher levels of glycine in the high SER, low ADC, and high SUV groups. Glycine consumption and synthesis is known to correlate with rapid cancer cell proliferation, and higher expression of the mitochondrial glycine biosynthesis pathway is associated with higher mortality in breast cancer patients [44]. Also, high levels of glycine have previously been shown to correlate with poor prognosis in breast cancer using HR-MAS [7, 26, 45, 46]. Previously, higher levels of glycine were found in ER negative and PR negative tumors than in ER positive and PR positive patients [26, 47], and higher levels of glycine were also associated with HER 2 overexpression regardless of hormone receptor status [26]. Along with PC and choline, well-known markers for breast cancer [7, 8], glycine can be a reliable marker in predicting treatment response in therapeutic monitoring or in suggesting poor prognosis for breast cancer patients.

ER, PR, HER-2, and Ki-67 expression have been shown to play a major role in breast cancer prognosis [48]. Metabolic differences caused by those hormone and growth receptors are highly relevant to tumor prognosis. However, the molecular reasons for their overexpression and amplification remain largely unknown. Previous reports have found some HR-MAS MRS values to be significantly correlated with known histopathologic prognostic factors [4, 26]. In the subgroup analysis in our study, multiple metabolites showed higher correlation with SER, ADC, and SUV in ER positive, PR positive, HER2 negative, and Ki-67 negative patients. In PLS-DA models for luminal subtype breast cancer, we observed a visual distinction between the high and low SER, ADC, and SUV groups. Also, high levels of glycine, PC, and choline were noted in the high SER, low ADC, and high SUV groups in luminal subtype breast cancer, indicating poor prognosis. Unlike the correlation study, the diagnostic performance of the multivariate profile analysis was not significantly better in the luminal subtype than in all cases, possibly because of our small sample size. Future studies with larger sample sizes might be able to detect the main metabolite in luminal subtype breast cancers that show typical high SER, low ADC, and high SUV image patterns, aiding in targeted therapy.

This study has several limitations. First, we included breast cancers of variable sizes, and 14 tumors (26.4%) were more than 3cm in diameter. Larger breast cancers show heterogeneous histologic features, which could cause variations in metabolite concentrations, angiogenesis, and tumor metabolism throughout the breast lesion. However, we were unable to compare HR-MAS MRS values obtained with CNB specimens with the spectroscopic values of the entire tumor volume. Therefore, our results might not fully represent the metabolic composition of large tumors. Second, the acquisition parameters in obtaining optimal spectral data for HR-MAS MR spectroscopy are important. There might have been minimal differences in each case, even though we tried to maintain the same acquisition parameters for each procedure. Third, we did not directly compare quantitative imaging parameters with breast cancer prognosis. When interpreting correlations with HR-MAS metabolite results and conventional imaging, we referred to previous reports that showed correlations between conventional imaging and prognostic factors. Our HR-MAS metabolic results might have limited explanatory power in predicting prognosis. Further studies should be constructed to compare different molecular subtypes of breast cancers with an increased number of cases and more survival data to validate our hypothesis. Fourth, we used a single relatively low b value for each lesion, either b = 600 sec/mm2 (n = 52) or b = 850 sec/mm2 (n = 1), to obtain ADC values. Therefore, we recommend caution in generalizing the lack of correlation between ADC values and breast cancer metabolites. However, this limitation might be of minimal effect because sensitivity and specificity are unaffected by the choice of b value even though different b values significantly affect the ADC of breast lesions, according to Dorrius et al. [49]. Lastly, we acquired our quantitative parameters using two different MR systems, which might have introduced heterogeneity into our data. We did find differences between the metabolite correlations with SER, and ADC when we analyzed the two groups separately. However, a large majority of ADC values were within 5% of values reported in previous studies that used different MR hardware and sequence parameters [50, 51], supporting the integration of ADC data in our study. A future study must be planned with a larger number of cases using one MR system for validation.

## Conclusion

High levels of PC, choline, and glycine acquired from HR-MAS MRS using CNB specimens were noted in the high SER group via DCE MRI and the high SUV group via PET-CT, with significant correlation between choline and SER and between PC and SUV. Further studies should investigate whether HR-MAS MRS using CNB specimens can provide similar or more prognostic information than conventional quantitative imaging parameters.

## Supporting Information

S1 Table Difference of HR-MAS MR spectroscopy metabolites between the high and low groups of conventional quantitative parameters (SER, ADC, SUV). (DOCX)

S2 Table HR-MAS MR spectroscopy values that showed significant correlation with SER, ADC, and SUV within the immunohistochemical groups. (DOCX)
