# Supplementary material for: Metabolomics of Breast Cancer Using High-Resolution Magic Angle Spinning Magnetic Resonance Spectroscopy: Correlations with 18F-FDG Positron Emission Tomography-Computed Tomography, Dynamic Contrast-Enhanced and Diffusion-Weighted Imaging MRI
Source: PLoS One. 2016 Jul 26;11(7):e0159949. doi: 10.1371/journal.pone.0159949 (PMC4961400; doi:10.1371/journal.pone.0159949)
Supplement: S1 Table — (DOCX) [file pone.0159949.s001.docx]

**S1 Table. Difference of HR-MAS MR spectroscopy metabolites between high and low groups of conventional quantitative parameters (SER, ADC, SUV).**

|  | Low SER (<0.96) | | High SER (0.96≤) | | P value | Low ADC (<1.38) | | High ADC (1.38≤) | | P value | Low SUV (<5.4) | | High SUV (5.4≤) | | P value |
| --- | --- | --- | --- | --- | --- | --- | --- | --- | --- | --- | --- | --- | --- | --- | --- |
|  | median | IQR | median | IQR |  | median | IQR | median | IQR |  | median | IQR | median | IQR |  |
| Acetate | 0.68 | 0.34 | 0.65 | 0.54 | 0.901 | 0.66 | 0.55 | 0.66 | 0.43 | 0.986 | 0.66 | 0.43 | 0.64 | 0.48 | 0.845 |
| Alanine | 5.46 | 2.50 | 5.22 | 3.26 | 0.423 | 4.90 | 3.01 | 6.26 | 2.65 | 0.165 | 6.26 | 2.65 | 5.43 | 2.51 | 0.762 |
| Arginine | 1.84 | 1.10 | 1.61 | 1.10 | 0.901 | 1.60 | 1.31 | 1.65 | 1.12 | 0.413 | 1.65 | 1.12 | 1.65 | 0.95 | 0.915 |
| Asparagine | 1.34 | 0.71 | 1.80 | 1.16 | **0.004** | 1.65 | 0.58 | 1.60 | 1.43 | 0.477 | 1.60 | 1.43 | 1.72 | 1.02 | **0.018** |
| Aspartate | 1.65 | 1.44 | 1.90 | 0.96 | 0.383 | 1.71 | 1.10 | 1.98 | 2.02 | 0.188 | 1.98 | 2.02 | 1.98 | 1.02 | 0.423 |
| Betaine | 0.30 | 0.21 | 0.28 | 0.29 | 0.943 | 0.29 | 0.23 | 0.30 | 0.30 | 0.618 | 0.30 | 0.30 | 0.28 | 0.28 | 0.722 |
| Choline | 1.69 | 0.77 | 1.86 | 0.99 | **0.019** | 1.77 | 1.19 | 1.71 | 0.54 | 0.709 | 1.71 | 0.54 | 1.79 | 0.46 | 0.278 |
| Creatine | 0.87 | 1.03 | 1.21 | 0.92 | 0.311 | 1.19 | 1.13 | 1.01 | 0.90 | 0.569 | 1.01 | 0.90 | 1.21 | 0.93 | 0.247 |
| Ethanol | 0.73 | 0.68 | 0.92 | 1.75 | 0.072 | 0.64 | 0.94 | 0.92 | 1.48 | 0.155 | 0.92 | 1.48 | 0.83 | 1.69 | 0.722 |
| Ethanolamine | 0.98 | 0.57 | 1.22 | 1.08 | 0.117 | 0.97 | 0.90 | 1.24 | 0.89 | 0.155 | 1.24 | 0.89 | 1.10 | 0.82 | 0.294 |
| Fumarate | 0.17 | 0.11 | 0.26 | 0.25 | **0.005** | 0.18 | 0.14 | 0.22 | 0.25 | **0.048** | 0.22 | 0.25 | 0.26 | 0.25 | **0.004** |
| Glucose | 4.56 | 3.13 | 3.76 | 1.82 | 0.355 | 4.09 | 2.11 | 3.86 | 2.83 | 0.929 | 3.86 | 2.83 | 3.90 | 1.50 | 0.762 |
| Glutamate | 6.87 | 2.38 | 7.01 | 4.38 | 0.488 | 6.22 | 2.77 | 8.09 | 3.21 | **0.009** | 8.09 | 3.21 | 7.30 | 2.64 | 0.364 |
| Glutamine | 1.39 | 1.20 | 1.79 | 0.96 | 0.150 | 1.95 | 1.00 | 1.59 | 0.99 | 0.466 | 1.59 | 0.99 | 2.00 | 0.97 | 0.160 |
| Glycerol | 1.69 | 1.08 | 2.00 | 1.33 | 0.545 | 1.88 | 1.09 | 1.74 | 1.71 | 0.631 | 1.74 | 1.71 | 2.02 | 1.23 | 0.084 |
| Glycine | 8.12 | 4.96 | 6.44 | 3.34 | 0.207 | 8.12 | 3.78 | 6.06 | 4.58 | 0.094 | 6.06 | 4.58 | 7.26 | 4.10 | 0.790 |
| Histidine | 0.53 | 0.45 | 0.72 | 0.41 | **0.001** | 0.63 | 0.32 | 0.63 | 0.40 | 0.915 | 0.63 | 0.40 | 0.64 | 0.33 | 0.346 |
| Isoleucine | 1.45 | 0.42 | 1.66 | 0.91 | 0.346 | 1.57 | 0.65 | 1.42 | 0.67 | 0.656 | 1.42 | 0.67 | 1.42 | 0.63 | 0.328 |
| Lactate | 11.23 | 8.52 | 11.44 | 7.89 | 0.831 | 10.80 | 8.58 | 11.44 | 6.20 | 0.618 | 11.44 | 6.20 | 11.71 | 7.59 | 0.374 |
| Leucine | 3.30 | 1.31 | 3.47 | 2.42 | 0.545 | 3.38 | 1.84 | 3.17 | 2.09 | 0.594 | 3.17 | 2.09 | 3.13 | 1.85 | 0.957 |
| Lysine | 2.10 | 1.20 | 2.77 | 4.46 | **0.044** | 2.72 | 2.12 | 2.29 | 1.81 | 0.575 | 2.29 | 1.81 | 2.74 | 1.91 | 0.072 |
| Methionine | 0.47 | 0.23 | 0.60 | 0.43 | 0.160 | 0.59 | 0.24 | 0.47 | 0.25 | 0.270 | 0.47 | 0.25 | 0.58 | 0.34 | 0.455 |
| PC | 1.10 | 1.05 | 1.43 | 1.11 | 0.150 | 1.34 | 1.00 | 1.18 | 1.33 | 0.644 | 1.18 | 1.33 | 1.58 | 1.08 | **0.046** |
| PE | 2.49 | 2.23 | 3.34 | 3.11 | 0.270 | 3.00 | 3.17 | 2.59 | 2.61 | 0.817 | 2.59 | 2.61 | 3.49 | 2.53 | **0.037** |
| Phenylalanine | 1.04 | 0.37 | 1.34 | 0.52 | **0.004** | 1.26 | 0.44 | 1.05 | 0.43 | 0.207 | 1.05 | 0.43 | 1.23 | 0.51 | 0.220 |
| Proline | 2.88 | 1.17 | 2.86 | 1.56 | 0.346 | 2.88 | 1.43 | 2.86 | 1.27 | 0.709 | 2.86 | 1.27 | 2.81 | 1.45 | 0.488 |
| Serine | 5.39 | 2.27 | 3.77 | 3.06 | 0.067 | 4.83 | 3.26 | 4.66 | 2.98 | 0.776 | 4.66 | 2.98 | 4.68 | 3.54 | 0.569 |
| Taurine | 5.21 | 3.72 | 4.42 | 3.35 | 0.278 | 5.36 | 4.09 | 4.62 | 3.24 | 0.240 | 4.62 | 3.24 | 5.03 | 3.33 | 0.817 |
| Threonine | 2.33 | 1.34 | 2.06 | 1.16 | 0.466 | 2.35 | 1.26 | 2.06 | 1.23 | 0.311 | 2.06 | 1.23 | 2.37 | 1.21 | 0.311 |
| Tyrosine | 1.14 | 0.35 | 1.33 | 0.83 | 0.008 | 1.29 | 0.46 | 1.18 | 0.37 | 0.557 | 1.18 | 0.37 | 1.30 | 0.50 | 0.255 |
| Uracil | 0.65 | 0.50 | 1.24 | 0.64 | **0.001** | 0.73 | 0.65 | 1.01 | 0.74 | 0.098 | 1.01 | 0.74 | 1.13 | 0.58 | **0.003** |
| Valine | 2.69 | 1.04 | 2.90 | 1.81 | 0.887 | 2.96 | 1.48 | 2.68 | 1.11 | 0.286 | 2.68 | 1.11 | 2.68 | 1.54 | 0.581 |
| myo-Inositol | 2.16 | 1.33 | 1.69 | 1.57 | 0.057 | 1.81 | 1.86 | 2.15 | 1.11 | 0.695 | 2.15 | 1.11 | 2.15 | 1.23 | 0.749 |
| GPC | 0.41 | 0.33 | 0.39 | 0.58 | 0.873 | 0.41 | 0.51 | 0.40 | 0.46 | 0.581 | 0.40 | 0.46 | 0.46 | 0.49 | 0.594 |

SER: signal enhancement ratio, SUV: standard uptake value, ADC: apparent diffusion coefficient

PC: Phosphocholine, PE: Phosphoethanolamine, GPC: Glycerophosphocholine
