# Supplementary material for: Metabolomics of Breast Cancer Using High-Resolution Magic Angle Spinning Magnetic Resonance Spectroscopy: Correlations with 18F-FDG Positron Emission Tomography-Computed Tomography, Dynamic Contrast-Enhanced and Diffusion-Weighted Imaging MRI
Source: PLoS One. 2016 Jul 26;11(7):e0159949. doi: 10.1371/journal.pone.0159949 (PMC4961400; doi:10.1371/journal.pone.0159949)
Supplement: S2 Table — (DOCX) [file pone.0159949.s002.docx]

**S2 Table. HR-MAS MR spectroscopy values that showed significant correlation with SER, ADC, and SUV within immunohistochemical groups.**

|  | | SER | | ADC | | SUV | |
| --- | --- | --- | --- | --- | --- | --- | --- |
|  |  | r^*^ | p-value | r^*^ | p-value | r^*^ | p-value |
| ER negative group (n=17) | | | | | | | |
|  | Acetate | 0.24 | 0.358 | -0.12 | 0.646 | 0.00 | 0.993 |
|  | Alanine | 0.10 | 0.694 | 0.00 | 0.989 | -0.04 | 0.877 |
|  | Arginine | 0.08 | 0.765 | 0.15 | 0.569 | -0.31 | 0.221 |
|  | Asparagine | 0.22 | 0.400 | 0.18 | 0.479 | 0.32 | 0.217 |
|  | Aspartate | 0.16 | 0.541 | 0.17 | 0.509 | -0.02 | 0.948 |
|  | Betaine | -0.12 | 0.646 | -0.03 | 0.896 | 0.32 | 0.215 |
|  | Choline | 0.13 | 0.613 | -0.14 | 0.592 | 0.08 | 0.764 |
|  | Creatine | 0.38 | 0.133 | -0.05 | 0.848 | 0.40 | 0.116 |
|  | Ethanol | -0.09 | 0.722 | 0.25 | 0.327 | -0.03 | 0.896 |
|  | Ethanolamine | -0.12 | 0.653 | 0.13 | 0.632 | -0.45 | 0.073 |
|  | Fumarate | -0.02 | 0.933 | 0.24 | 0.357 | 0.30 | 0.237 |
|  | Glucose | 0.09 | 0.736 | -0.05 | 0.841 | -0.42 | 0.094 |
|  | Glutamate | 0.13 | 0.613 | 0.15 | 0.563 | -0.02 | 0.925 |
|  | Glutamine | 0.24 | 0.353 | 0.05 | 0.852 | 0.16 | 0.534 |
|  | Glycerol | 0.21 | 0.428 | 0.19 | 0.461 | 0.07 | 0.786 |
|  | Glycine | 0.37 | 0.149 | 0.02 | 0.940 | -0.09 | 0.722 |
|  | Histidine | 0.24 | 0.348 | -0.04 | 0.881 | 0.32 | 0.212 |
|  | Isoleucine | 0.21 | 0.428 | 0.05 | 0.859 | -0.25 | 0.335 |
|  | Lactate | -0.16 | 0.529 | 0.17 | 0.506 | 0.10 | 0.701 |
|  | Leucine | 0.08 | 0.772 | 0.00 | 0.985 | -0.21 | 0.430 |
|  | Lysine | 0.35 | 0.168 | -0.05 | 0.841 | -0.02 | 0.952 |
|  | Methionine | 0.45 | 0.069 | 0.13 | 0.609 | -0.22 | 0.391 |
|  | PC | 0.35 | 0.165 | 0.18 | 0.500 | 0.47 | 0.055 |
|  | PE | 0.35 | 0.174 | -0.08 | 0.768 | 0.28 | 0.274 |
|  | Phenylalanine | 0.35 | 0.171 | -0.11 | 0.687 | 0.15 | 0.563 |
|  | Proline | 0.00 | 0.993 | 0.00 | 0.989 | -0.13 | 0.615 |
|  | Serine | 0.22 | 0.390 | 0.03 | 0.922 | -0.31 | 0.233 |
|  | Taurine | -0.05 | 0.859 | -0.09 | 0.739 | 0.03 | 0.911 |
|  | Threonine | 0.14 | 0.580 | -0.27 | 0.294 | -0.25 | 0.337 |
|  | Tyrosine | 0.34 | 0.188 | 0.21 | 0.424 | 0.15 | 0.560 |
|  | Uracil | 0.26 | 0.319 | 0.07 | 0.793 | 0.09 | 0.718 |
|  | Valine | 0.18 | 0.480 | -0.04 | 0.877 | -0.16 | 0.537 |
|  | myo-Inositol | 0.16 | 0.541 | 0.40 | 0.109 | 0.04 | 0.885 |
|  | GPC | -0.09 | 0.729 | 0.04 | 0.866 | 0.23 | 0.383 |
| ER positive group (n=36) | | | | | | | |
|  | Acetate | -0.06 | 0.725 | 0.19 | 0.255 | 0.14 | 0.430 |
|  | Alanine | -0.14 | 0.422 | 0.26 | 0.131 | -0.01 | 0.958 |
|  | Arginine | 0.11 | 0.542 | 0.04 | 0.814 | 0.04 | 0.795 |
|  | Asparagine | **0.43** | **0.009** | 0.00 | 0.983 | 0.31 | 0.065 |
|  | Aspartate | 0.10 | 0.564 | 0.11 | 0.512 | 0.06 | 0.730 |
|  | Betaine | -0.09 | 0.589 | 0.20 | 0.246 | 0.14 | 0.407 |
|  | Choline | 0.30 | 0.071 | -0.08 | 0.635 | 0.18 | 0.306 |
|  | Creatine | 0.06 | 0.718 | -0.10 | 0.561 | 0.20 | 0.241 |
|  | Ethanol | **0.34** | **0.041** | 0.06 | 0.730 | -0.05 | 0.783 |
|  | Ethanolamine | **0.40** | **0.016** | 0.21 | 0.223 | 0.22 | 0.202 |
|  | Fumarate | **0.53** | **0.001** | 0.14 | 0.429 | **0.43** | **0.010** |
|  | Glucose | -0.05 | 0.763 | -0.05 | 0.788 | 0.05 | 0.757 |
|  | Glutamate | 0.18 | 0.299 | 0.14 | 0.427 | 0.19 | 0.264 |
|  | Glutamine | **0.36** | **0.030** | -0.04 | 0.807 | 0.24 | 0.153 |
|  | Glycerol | 0.04 | 0.815 | -0.21 | 0.210 | 0.16 | 0.356 |
|  | Glycine | -0.08 | 0.660 | -0.32 | 0.057 | 0.21 | 0.229 |
|  | Histidine | **0.61** | **0.000** | -0.11 | 0.530 | 0.18 | 0.306 |
|  | Isoleucine | 0.16 | 0.361 | 0.14 | 0.406 | -0.16 | 0.350 |
|  | Lactate | 0.01 | 0.956 | -0.08 | 0.626 | **0.34** | **0.042** |
|  | Leucine | 0.15 | 0.375 | 0.18 | 0.299 | -0.01 | 0.949 |
|  | Lysine | **0.38** | **0.022** | -0.03 | 0.882 | 0.23 | 0.175 |
|  | Methionine | 0.25 | 0.142 | 0.05 | 0.773 | 0.16 | 0.357 |
|  | PC | 0.25 | 0.136 | -0.20 | 0.238 | 0.32 | 0.053 |
|  | PE | 0.17 | 0.312 | -0.18 | 0.280 | 0.24 | 0.155 |
|  | Phenylalanine | **0.44** | **0.008** | -0.11 | 0.541 | 0.05 | 0.751 |
|  | Proline | 0.19 | 0.260 | -0.18 | 0.289 | 0.15 | 0.375 |
|  | Serine | -0.16 | 0.345 | -0.08 | 0.664 | -0.04 | 0.839 |
|  | Taurine | 0.00 | 0.989 | -0.30 | 0.079 | 0.05 | 0.755 |
|  | Threonine | 0.05 | 0.784 | -0.22 | 0.198 | 0.16 | 0.357 |
|  | Tyrosine | **0.45** | **0.007** | -0.03 | 0.875 | 0.16 | 0.366 |
|  | Uracil | **0.61** | **0.000** | 0.04 | 0.803 | **0.54** | **0.001** |
|  | Valine | 0.00 | 0.992 | 0.07 | 0.703 | 0.00 | 0.991 |
|  | myo-Inositol | -0.06 | 0.709 | -0.30 | 0.075 | 0.05 | 0.763 |
|  | GPC | -0.12 | 0.488 | 0.09 | 0.605 | -0.05 | 0.793 |
| PR negative group (n=37) | | | | | | | |
|  | Acetate | 0.09 | 0.600 | 0.31 | 0.062 | 0.12 | 0.467 |
|  | Alanine | 0.08 | 0.644 | 0.16 | 0.344 | 0.05 | 0.781 |
|  | Arginine | 0.22 | 0.185 | 0.21 | 0.208 | -0.01 | 0.963 |
|  | Asparagine | **0.38** | **0.021** | 0.31 | 0.060 | **0.36** | **0.029** |
|  | Aspartate | 0.27 | 0.103 | **0.34** | **0.043** | 0.27 | 0.109 |
|  | Betaine | -0.27 | 0.101 | 0.13 | 0.451 | 0.14 | 0.416 |
|  | Choline | 0.16 | 0.343 | 0.01 | 0.949 | 0.05 | 0.758 |
|  | Creatine | 0.26 | 0.119 | -0.01 | 0.974 | 0.25 | 0.142 |
|  | Ethanol | 0.01 | 0.953 | 0.06 | 0.719 | -0.19 | 0.252 |
|  | Ethanolamine | 0.26 | 0.115 | 0.29 | 0.078 | 0.03 | 0.839 |
|  | Fumarate | 0.19 | 0.260 | 0.22 | 0.190 | 0.29 | 0.084 |
|  | Glucose | 0.27 | 0.103 | -0.01 | 0.946 | 0.17 | 0.320 |
|  | Glutamate | 0.32 | 0.051 | 0.25 | 0.142 | 0.26 | 0.115 |
|  | Glutamine | 0.28 | 0.094 | 0.08 | 0.652 | 0.09 | 0.610 |
|  | Glycerol | 0.25 | 0.134 | 0.01 | 0.952 | 0.30 | 0.071 |
|  | Glycine | 0.14 | 0.403 | -0.21 | 0.217 | 0.21 | 0.222 |
|  | Histidine | **0.35** | **0.036** | 0.10 | 0.545 | 0.14 | 0.415 |
|  | Isoleucine | 0.15 | 0.380 | 0.17 | 0.321 | -0.19 | 0.261 |
|  | Lactate | -0.05 | 0.786 | -0.09 | 0.585 | 0.29 | 0.087 |
|  | Leucine | 0.08 | 0.622 | 0.21 | 0.203 | -0.03 | 0.859 |
|  | Lysine | 0.20 | 0.236 | 0.04 | 0.831 | 0.16 | 0.352 |
|  | Methionine | 0.22 | 0.191 | 0.18 | 0.290 | 0.02 | 0.928 |
|  | PC | 0.19 | 0.263 | 0.02 | 0.925 | 0.29 | 0.086 |
|  | PE | 0.29 | 0.078 | 0.00 | 0.999 | **0.45** | **0.005** |
|  | Phenylalanine | **0.42** | **0.010** | 0.05 | 0.772 | 0.12 | 0.471 |
|  | Proline | 0.17 | 0.306 | -0.02 | 0.897 | 0.13 | 0.455 |
|  | Serine | 0.25 | 0.140 | 0.10 | 0.550 | 0.17 | 0.313 |
|  | Taurine | -0.02 | 0.917 | -0.20 | 0.238 | 0.08 | 0.627 |
|  | Threonine | 0.09 | 0.616 | -0.13 | 0.449 | 0.23 | 0.169 |
|  | Tyrosine | **0.35** | **0.037** | 0.26 | 0.126 | 0.14 | 0.393 |
|  | Uracil | **0.34** | **0.040** | 0.20 | 0.236 | **0.38** | **0.019** |
|  | Valine | -0.03 | 0.863 | 0.11 | 0.510 | -0.09 | 0.617 |
|  | myo-Inositol | 0.15 | 0.368 | -0.03 | 0.868 | 0.17 | 0.303 |
|  | GPC | -0.12 | 0.463 | 0.08 | 0.651 | -0.02 | 0.898 |
| PR positive group (n=16) | | | | | | | |
|  | Acetate | 0.15 | 0.579 | -0.37 | 0.154 | 0.05 | 0.850 |
|  | Alanine | -0.34 | 0.204 | 0.35 | 0.185 | -0.21 | 0.427 |
|  | Arginine | -0.19 | 0.485 | -0.25 | 0.347 | 0.04 | 0.884 |
|  | Asparagine | 0.37 | 0.161 | -0.36 | 0.174 | -0.01 | 0.970 |
|  | Aspartate | 0.01 | 0.983 | -0.36 | 0.166 | -0.27 | 0.305 |
|  | Betaine | -0.21 | 0.444 | -0.05 | 0.854 | 0.00 | 0.996 |
|  | Choline | 0.39 | 0.131 | -0.25 | 0.356 | 0.03 | 0.914 |
|  | Creatine | 0.17 | 0.528 | -0.07 | 0.807 | 0.31 | 0.247 |
|  | Ethanol | 0.06 | 0.829 | 0.05 | 0.858 | 0.16 | 0.557 |
|  | Ethanolamine | 0.29 | 0.269 | -0.24 | 0.380 | -0.22 | 0.408 |
|  | Fumarate | **0.58** | **0.019** | 0.09 | 0.733 | 0.40 | 0.129 |
|  | Glucose | -0.22 | 0.412 | 0.12 | 0.648 | -0.20 | 0.468 |
|  | Glutamate | -0.22 | 0.412 | 0.29 | 0.276 | -0.09 | 0.745 |
|  | Glutamine | 0.23 | 0.387 | -0.28 | 0.297 | 0.33 | 0.217 |
|  | Glycerol | 0.16 | 0.542 | -0.07 | 0.803 | 0.01 | 0.978 |
|  | Glycine | 0.07 | 0.803 | -0.35 | 0.178 | -0.01 | 0.961 |
|  | Histidine | 0.36 | 0.169 | **-0.61** | **0.012** | -0.17 | 0.520 |
|  | Isoleucine | 0.05 | 0.846 | -0.27 | 0.310 | -0.45 | 0.078 |
|  | Lactate | 0.10 | 0.721 | 0.41 | 0.113 | **0.57** | **0.022** |
|  | Leucine | 0.11 | 0.680 | -0.28 | 0.302 | -0.38 | 0.149 |
|  | Lysine | **0.55** | **0.028** | -0.27 | 0.305 | -0.07 | 0.807 |
|  | Methionine | 0.34 | 0.196 | -0.38 | 0.150 | 0.03 | 0.901 |
|  | PC | 0.41 | 0.119 | -0.23 | 0.392 | 0.24 | 0.365 |
|  | PE | 0.34 | 0.192 | -0.31 | 0.239 | 0.10 | 0.704 |
|  | Phenylalanine | 0.25 | 0.345 | **-0.50** | **0.047** | -0.24 | 0.377 |
|  | Proline | 0.28 | 0.295 | -0.16 | 0.546 | 0.27 | 0.305 |
|  | Serine | -0.15 | 0.587 | -0.23 | 0.386 | -0.35 | 0.185 |
|  | Taurine | 0.03 | 0.905 | -0.36 | 0.168 | -0.13 | 0.637 |
|  | Threonine | 0.36 | 0.176 | -0.37 | 0.159 | -0.11 | 0.688 |
|  | Tyrosine | 0.44 | 0.087 | **-0.52** | **0.038** | -0.08 | 0.782 |
|  | Uracil | 0.45 | 0.078 | -0.07 | 0.807 | 0.41 | 0.117 |
|  | Valine | 0.10 | 0.721 | -0.32 | 0.223 | -0.29 | 0.268 |
|  | myo-Inositol | -0.19 | 0.485 | -0.07 | 0.787 | -0.29 | 0.268 |
|  | GPC | 0.01 | 0.983 | 0.14 | 0.606 | 0.28 | 0.286 |
| HER2 negative group (n=41) | | | | | | | |
|  | Acetate | -0.01 | 0.966 | -0.04 | 0.813 | -0.06 | 0.715 |
|  | Alanine | -0.15 | 0.349 | 0.13 | 0.434 | -0.11 | 0.503 |
|  | Arginine | -0.01 | 0.934 | -0.04 | 0.812 | -0.10 | 0.544 |
|  | Asparagine | **0.46** | **0.003** | 0.12 | 0.470 | **0.42** | **0.007** |
|  | Aspartate | 0.22 | 0.175 | 0.08 | 0.637 | 0.16 | 0.326 |
|  | Betaine | -0.11 | 0.486 | 0.08 | 0.640 | 0.00 | 0.997 |
|  | Choline | **0.37** | **0.019** | 0.05 | 0.741 | **0.32** | **0.040** |
|  | Creatine | 0.14 | 0.384 | -0.21 | 0.191 | 0.16 | 0.306 |
|  | Ethanol | 0.20 | 0.207 | 0.20 | 0.211 | 0.08 | 0.638 |
|  | Ethanolamine | **0.33** | **0.033** | 0.04 | 0.796 | 0.09 | 0.589 |
|  | Fumarate | **0.41** | **0.007** | 0.18 | 0.252 | **0.46** | **0.002** |
|  | Glucose | 0.01 | 0.940 | -0.05 | 0.764 | -0.03 | 0.848 |
|  | Glutamate | .317^*^ | 0.043 | 0.18 | 0.253 | **0.35** | **0.026** |
|  | Glutamine | 0.24 | 0.127 | -0.05 | 0.745 | 0.14 | 0.386 |
|  | Glycerol | 0.15 | 0.345 | -0.04 | 0.801 | 0.21 | 0.179 |
|  | Glycine | -0.01 | 0.927 | -0.30 | 0.060 | 0.03 | 0.859 |
|  | Histidine | **0.50** | **0.001** | -0.06 | 0.695 | 0.23 | 0.153 |
|  | Isoleucine | -0.04 | 0.792 | -0.04 | 0.807 | **-0.40** | **0.009** |
|  | Lactate | 0.21 | 0.181 | 0.24 | 0.125 | **0.43** | **0.005** |
|  | Leucine | -0.03 | 0.867 | 0.00 | 0.980 | -0.21 | 0.192 |
|  | Lysine | 0.14 | 0.370 | -0.14 | 0.386 | 0.01 | 0.933 |
|  | Methionine | 0.14 | 0.377 | -0.12 | 0.442 | -0.18 | 0.249 |
|  | PC | 0.21 | 0.186 | -0.15 | 0.357 | 0.27 | 0.089 |
|  | PE | **0.31** | **0.047** | -0.19 | 0.235 | **0.33** | **0.038** |
|  | Phenylalanine | 0.23 | 0.151 | -0.24 | 0.133 | -0.05 | 0.736 |
|  | Proline | 0.26 | 0.105 | -0.11 | 0.478 | 0.15 | 0.344 |
|  | Serine | -0.13 | 0.430 | -0.13 | 0.410 | -0.21 | 0.198 |
|  | Taurine | -0.02 | 0.889 | -0.26 | 0.102 | 0.01 | 0.947 |
|  | Threonine | 0.07 | 0.675 | **-0.41** | **0.008** | -0.09 | 0.588 |
|  | Tyrosine | 0.30 | 0.053 | -0.13 | 0.435 | 0.01 | 0.975 |
|  | Uracil | **0.55** | **0.000** | 0.01 | 0.927 | **0.53** | **0.000** |
|  | Valine | -0.10 | 0.522 | -0.06 | 0.710 | -0.28 | 0.077 |
|  | myo-Inositol | -0.20 | 0.204 | -0.12 | 0.469 | -0.10 | 0.526 |
|  | GPC | -0.05 | 0.735 | 0.13 | 0.433 | 0.03 | 0.871 |
| HER2 positive group (n=12) | | | | | | | |
|  | Acetate | -0.08 | 0.812 | 0.51 | 0.087 | 0.44 | 0.152 |
|  | Alanine | 0.19 | 0.557 | 0.22 | 0.498 | 0.08 | 0.812 |
|  | Arginine | 0.57 | 0.051 | 0.17 | 0.601 | 0.15 | 0.649 |
|  | Asparagine | 0.29 | 0.354 | 0.05 | 0.871 | 0.14 | 0.665 |
|  | Aspartate | 0.18 | 0.572 | 0.34 | 0.285 | -0.03 | 0.914 |
|  | Betaine | -0.36 | 0.255 | 0.26 | 0.409 | **0.70** | **0.011** |
|  | Choline | -0.18 | 0.572 | -0.52 | 0.082 | -0.36 | 0.245 |
|  | Creatine | 0.40 | 0.199 | 0.26 | 0.409 | **0.66** | **0.020** |
|  | Ethanol | 0.13 | 0.697 | 0.42 | 0.178 | -0.18 | 0.572 |
|  | Ethanolamine | 0.01 | 0.983 | **0.58** | **0.049** | -0.10 | 0.746 |
|  | Fumarate | 0.03 | 0.914 | 0.41 | 0.182 | -0.14 | 0.665 |
|  | Glucose | 0.00 | 1.000 | 0.01 | 0.974 | -0.01 | 0.983 |
|  | Glutamate | 0.29 | 0.354 | 0.39 | 0.212 | -0.20 | 0.542 |
|  | Glutamine | 0.08 | 0.795 | -0.33 | 0.301 | 0.16 | 0.618 |
|  | Glycerol | 0.12 | 0.713 | -0.04 | 0.905 | 0.22 | 0.499 |
|  | Glycine | -0.30 | 0.342 | -0.19 | 0.549 | -0.04 | 0.897 |
|  | Histidine | 0.18 | 0.572 | 0.19 | 0.549 | 0.08 | 0.812 |
|  | Isoleucine | 0.39 | 0.208 | 0.12 | 0.721 | 0.14 | 0.665 |
|  | Lactate | -0.36 | 0.245 | **-0.61** | **0.037** | -0.10 | 0.746 |
|  | Leucine | 0.05 | 0.880 | 0.34 | 0.280 | 0.11 | 0.729 |
|  | Lysine | -0.10 | 0.762 | 0.13 | 0.680 | 0.35 | 0.265 |
|  | Methionine | 0.19 | 0.557 | 0.30 | 0.341 | 0.50 | 0.095 |
|  | PC | 0.09 | 0.779 | 0.42 | 0.170 | 0.23 | 0.471 |
|  | PE | 0.08 | 0.812 | 0.12 | 0.721 | 0.54 | 0.071 |
|  | Phenylalanine | 0.52 | 0.080 | 0.34 | 0.280 | 0.11 | 0.729 |
|  | Proline | -0.36 | 0.245 | -0.05 | 0.871 | 0.21 | 0.513 |
|  | Serine | 0.08 | 0.812 | -0.02 | 0.940 | 0.10 | 0.762 |
|  | Taurine | -0.27 | 0.391 | -0.51 | 0.092 | -0.24 | 0.443 |
|  | Threonine | -0.48 | 0.112 | -0.04 | 0.905 | 0.28 | 0.379 |
|  | Tyrosine | 0.29 | 0.354 | 0.49 | 0.103 | 0.36 | 0.255 |
|  | Uracil | 0.15 | 0.633 | **0.74** | **0.006** | 0.02 | 0.948 |
|  | Valine | -0.20 | 0.527 | 0.10 | 0.762 | 0.44 | 0.152 |
|  | myo-Inositol | 0.30 | 0.342 | -0.03 | 0.931 | -0.15 | 0.633 |
|  | GPC | -0.23 | 0.471 | -0.06 | 0.854 | 0.22 | 0.499 |
| Ki67 negative group (n=22) | | | | | | | |
|  | Acetate | -0.10 | 0.651 | 0.29 | 0.187 | 0.34 | 0.116 |
|  | Alanine | **-0.45** | **0.037** | 0.32 | 0.142 | -0.17 | 0.460 |
|  | Arginine | -0.05 | 0.836 | 0.03 | 0.895 | -0.08 | 0.740 |
|  | Asparagine | 0.41 | 0.058 | 0.12 | 0.604 | **0.50** | **0.018** |
|  | Aspartate | 0.26 | 0.246 | -0.11 | 0.612 | 0.17 | 0.445 |
|  | Betaine | 0.00 | 0.986 | -0.01 | 0.966 | 0.12 | 0.602 |
|  | Choline | 0.25 | 0.259 | 0.12 | 0.584 | 0.41 | 0.059 |
|  | Creatine | -0.14 | 0.543 | -0.02 | 0.923 | 0.22 | 0.314 |
|  | Ethanol | 0.31 | 0.159 | 0.05 | 0.820 | 0.03 | 0.891 |
|  | Ethanolamine | 0.31 | 0.167 | 0.28 | 0.208 | 0.20 | 0.382 |
|  | Fumarate | **0.48** | **0.023** | 0.19 | 0.398 | **0.54** | **0.009** |
|  | Glucose | -0.12 | 0.597 | -0.05 | 0.820 | -0.08 | 0.726 |
|  | Glutamate | 0.14 | 0.533 | -0.07 | 0.751 | 0.18 | 0.425 |
|  | Glutamine | 0.36 | 0.105 | 0.17 | 0.456 | 0.31 | 0.164 |
|  | Glycerol | 0.24 | 0.275 | -0.13 | 0.553 | 0.29 | 0.193 |
|  | Glycine | -0.08 | 0.721 | -0.39 | 0.076 | 0.19 | 0.390 |
|  | Histidine | **0.61** | **0.003** | -0.15 | 0.514 | 0.18 | 0.415 |
|  | Isoleucine | 0.21 | 0.357 | 0.36 | 0.105 | -0.13 | 0.563 |
|  | Lactate | 0.17 | 0.440 | -0.15 | 0.506 | **0.44** | **0.042** |
|  | Leucine | 0.08 | 0.732 | **0.46** | **0.032** | -0.06 | 0.795 |
|  | Lysine | **0.51** | **0.015** | 0.21 | 0.345 | 0.17 | 0.444 |
|  | Methionine | 0.27 | 0.216 | 0.27 | 0.230 | 0.05 | 0.814 |
|  | PC | 0.12 | 0.608 | -0.41 | 0.056 | 0.20 | 0.365 |
|  | PE | 0.25 | 0.266 | -0.36 | 0.105 | 0.16 | 0.484 |
|  | Phenylalanine | 0.34 | 0.120 | -0.02 | 0.930 | 0.02 | 0.919 |
|  | Proline | **0.48** | **0.024** | -0.15 | 0.497 | 0.18 | 0.418 |
|  | Serine | -0.16 | 0.471 | -0.13 | 0.559 | -0.03 | 0.883 |
|  | Taurine | -0.03 | 0.883 | **-0.63** | **0.002** | -0.08 | 0.736 |
|  | Threonine | 0.09 | 0.695 | -0.16 | 0.471 | 0.12 | 0.582 |
|  | Tyrosine | 0.37 | 0.087 | 0.18 | 0.434 | 0.17 | 0.452 |
|  | Uracil | **0.64** | **0.001** | -0.02 | 0.930 | **0.73** | **0.000** |
|  | Valine | 0.02 | 0.938 | 0.20 | 0.371 | -0.01 | 0.950 |
|  | myo-Inositol | -0.03 | 0.907 | -0.24 | 0.287 | -0.04 | 0.863 |
|  | GPC | -0.13 | 0.556 | -0.03 | 0.883 | -0.07 | 0.762 |
| Ki67 positive group (n=29) | | | | | | | |
|  | Acetate | 0.19 | 0.335 | -0.01 | 0.951 | -0.05 | 0.803 |
|  | Alanine | 0.10 | 0.624 | 0.15 | 0.436 | 0.00 | 0.990 |
|  | Arginine | 0.24 | 0.205 | 0.03 | 0.857 | -0.08 | 0.664 |
|  | Asparagine | 0.24 | 0.201 | 0.00 | 0.981 | 0.14 | 0.455 |
|  | Aspartate | 0.10 | 0.606 | 0.19 | 0.320 | -0.03 | 0.883 |
|  | Betaine | -0.26 | 0.176 | 0.19 | 0.326 | 0.23 | 0.234 |
|  | Choline | 0.10 | 0.599 | -0.34 | 0.072 | -0.12 | 0.550 |
|  | Creatine | 0.25 | 0.199 | -0.13 | 0.490 | 0.07 | 0.734 |
|  | Ethanol | 0.01 | 0.964 | 0.22 | 0.253 | -0.18 | 0.350 |
|  | Ethanolamine | 0.10 | 0.618 | -0.05 | 0.789 | -0.32 | 0.095 |
|  | Fumarate | 0.05 | 0.780 | 0.11 | 0.584 | 0.14 | 0.473 |
|  | Glucose | 0.09 | 0.640 | -0.11 | 0.581 | -0.13 | 0.494 |
|  | Glutamate | 0.10 | 0.597 | 0.33 | 0.080 | 0.01 | 0.948 |
|  | Glutamine | 0.23 | 0.238 | -0.19 | 0.333 | 0.12 | 0.544 |
|  | Glycerol | 0.01 | 0.949 | -0.16 | 0.412 | 0.03 | 0.894 |
|  | Glycine | 0.06 | 0.739 | -0.26 | 0.167 | -0.05 | 0.798 |
|  | Histidine | 0.26 | 0.180 | -0.10 | 0.592 | 0.03 | 0.869 |
|  | Isoleucine | 0.26 | 0.181 | -0.03 | 0.858 | -0.21 | 0.280 |
|  | Lactate | -0.20 | 0.302 | 0.14 | 0.483 | 0.24 | 0.208 |
|  | Leucine | 0.17 | 0.371 | -0.10 | 0.600 | -0.01 | 0.940 |
|  | Lysine | 0.14 | 0.479 | -0.30 | 0.109 | 0.10 | 0.622 |
|  | Methionine | 0.36 | 0.057 | -0.18 | 0.339 | -0.01 | 0.954 |
|  | PC | 0.17 | 0.371 | 0.14 | 0.481 | 0.28 | 0.147 |
|  | PE | 0.10 | 0.624 | -0.02 | 0.919 | 0.22 | 0.262 |
|  | Phenylalanine | **0.43** | **0.020** | -0.14 | 0.478 | 0.11 | 0.580 |
|  | Proline | -0.10 | 0.592 | -0.09 | 0.625 | 0.19 | 0.326 |
|  | Serine | 0.00 | 0.992 | -0.11 | 0.557 | -0.17 | 0.365 |
|  | Taurine | -0.08 | 0.670 | -0.03 | 0.883 | 0.13 | 0.499 |
|  | Threonine | -0.03 | 0.871 | -0.33 | 0.076 | 0.04 | 0.847 |
|  | Tyrosine | 0.30 | 0.120 | -0.09 | 0.635 | 0.03 | 0.866 |
|  | Uracil | 0.12 | 0.538 | 0.01 | 0.952 | 0.03 | 0.857 |
|  | Valine | 0.09 | 0.647 | -0.12 | 0.539 | -0.07 | 0.715 |
|  | myo-Inositol | -0.03 | 0.891 | -0.05 | 0.783 | 0.03 | 0.890 |
|  | GPC | -0.09 | 0.658 | 0.18 | 0.357 | 0.23 | 0.235 |

r^*^: Spearman correlation coefficient.

ER: estrogen receptor, PR: progesterone receptor, HER2: human epidermal growth factor receptor

SER: signal enhancement ratio, SUV: standard uptake value, ADC: apparent diffusion coefficient

PC: Phosphocholine, PE: Phosphoethanolamine, GPC: Glycerophosphocholine
